# Effects of a Pharmacist–Physician Collaborative Protocol for Acute Blood Pressure Management of Patients with Intracerebral Hemorrhage

**DOI:** 10.3390/jcm15218733

**Published:** 2026-11-09

**Authors:** Takaya Sagawa, Hitoshi Fukuda, Tomoaki Ishida, Ryota Sakoda, Shumpei Morisawa, Keita Akagaki, Takumi Maruyama, Kei Kawada, Ken Shinno, Takako Kawamura, Yu Kawanishi, Tetsuya Ueba, Yukihiro Hamada, Kohei Jobu

**Affiliations:** 1 Department of Pharmacy, Kochi Medical School Hospital, Kochi University, 185-1, Kohasu, Oko-cho, Nankoku-shi 7838505, Kochi, Japan; jm-tky-s@kochi-u.ac.jp (T.S.); jm-rsakoda@kochi-u.ac.jp (R.S.); jm-morisawa.s@kochi-u.ac.jp (S.M.); jm-keita_akagaki@kochi-u.ac.jp (K.A.); jm-maruyama.takumi@kochi-u.ac.jp (T.M.); hamada_yukihiro@kochi-u.ac.jp (Y.H.); jm-kouheij@kochi-u.ac.jp (K.J.); 2 Graduate School of Integrated Arts and Sciences, Kochi University, 185-1, Kohasu, Oko-cho, Nankoku-shi 7838505, Kochi, Japan; 3 Department of Neurosurgery, Kochi Medical School, Kochi University, 185-1, Kohasu, Oko-cho, Nankoku-shi 7838505, Kochi, Japan; k.shinno.1017@gmail.com (K.S.); pukutaka777@gmail.com (T.K.); ykawanishi@kochi-u.ac.jp (Y.K.); tueba@kochi-u.ac.jp (T.U.); 4 Department of Clinical Pharmacy, Graduate School of Pharmaceutical Sciences, Nagoya City University, 3-1 Tanabe-dori, Mizuho-ku, Nagoya-shi 4678603, Aichi, Japan; t-ishida@phar.nagoya-cu.ac.jp; 5 Department of Clinical Pharmacology and Therapeutics, Graduate School of Biomedical Sciences, Tokushima University, 3-18-15 Kuramoto-cho, Tokushima 7708503, Tokushima, Japan; kawada.kei@tokushima-u.ac.jp; 6 Department of Pharmacy, Tokushima University Hospital, 2-50-1 Kuramoto-cho, Tokushima 7708503, Tokushima, Japan

**Keywords:** intracerebral hemorrhage (ICH), blood pressure management, nicardipine, pharmacist–physician collaborative protocol

## Abstract

**Background/Objectives:** In the acute and subacute phases of spontaneous intracerebral hemorrhage (ICH), appropriate systolic blood pressure (SBP) control is important to limit hematoma expansion. However, optimal blood pressure management should be individualized according to hemorrhage severity and the patient’s clinical condition. In clinical practice, dose control of antihypertensive agents and timing of transition from intravenous to oral agents have been left at the discretion of attending physicians, resulting in fluctuations in blood pressure and delayed withdrawal from intravenous agents. In this study, pharmacists and physicians collaborated to develop a standardized blood pressure management protocol for acute ICH to achieve the target SBP and facilitate early withdrawal from intravenous antihypertensive agents. **Methods:** In March 2022, we developed a protocol comprising strict blood pressure control during the first 24 h, standardized titration of intravenous antihypertensive agents, and predefined oral antihypertensive therapy. We evaluated the effects of the protocol on achievement of SBP < 140 mmHg over 7 days, timing of withdrawal from intravenous antihypertensive agents, and unfavorable consequences associated with prolonged intravenous infusion, including delirium. **Results:** Achievement of SBP < 140 mmHg significantly increased from 20% (9/46) to 54% (27/50) after protocol implementation (*p* = 0.001), with a significant adjusted association (adjusted odds ratio, 5.51; 95% CI, 2.09–14.51). Median time to nicardipine discontinuation decreased from 72.5 to 37.5 h (log-rank *p* = 0.018). Delirium incidence was unchanged, whereas its median duration among affected patients decreased from 2 to 1 day. **Conclusions:** Protocol implementation was associated with improved target SBP achievement and earlier intravenous nicardipine discontinuation. Clinical benefits and safety require further investigation.

## 1. Introduction

Despite recent advances in drug therapy and surgical treatment, intracerebral hemorrhage (ICH) remains a disease with high morbidity and mortality [1]. One of the key factors determining the prognosis of patients with ICH is hematoma expansion occurring in the early post-onset period; as such, preventing this expansion is crucial for improving outcomes [2]. Previous clinical studies and guidelines have suggested that appropriate blood pressure reduction during the early stage of the disease may help limit hematoma expansion [2,3,4]. However, current guidelines recommend careful blood pressure management in selected patients with ICH and caution against excessive acute blood pressure reduction, particularly to systolic blood pressure (SBP) levels below 130 mmHg. Intravenous antihypertensive agents, including calcium channel blockers, have been recommended to rapidly achieve the target systolic blood pressure (SBP) in the acute phase, followed by a transition to oral antihypertensive drugs in the subacute phase for long-term blood pressure control [2,4]. However, in clinical practice, dose control of antihypertensive agents and the timing of transition from intravenous to oral drugs are not clearly defined and thus commonly depend on the discretion of the attending physicians. Consequently, variations in blood pressure control and delays in the transition to oral antihypertensive drugs may occur. Prolonged administration of intravenous antihypertensive drugs may restrict patients’ activity and mobility and potentially contribute to complications such as disuse syndrome and delirium [5,6]. Pharmacists may contribute to the standardization of antihypertensive management through medication reconciliation, assessment of preadmission antihypertensive therapy, evaluation of contraindications and drug interactions, monitoring of renal function and electrolytes, and recommendations regarding dose adjustments and the transition from intravenous to oral antihypertensive agents. These activities are performed in collaboration with physicians, who retain responsibility for prescribing decisions. We recently developed a novel protocol for blood pressure control in patients with acute ICH, in which pharmacists and physicians collaborated to provide standardized treatment by focusing on dose control, timing of transition, and choice of antihypertensive drugs. In the present study, we evaluated whether the novel protocol contributed to the achievement of the target SBP and earlier withdrawal from intravenous antihypertensive drugs. The protocol incorporated standardized intravenous nicardipine titration and predefined oral antihypertensive treatment. In the present study, we evaluated the association between implementation of this collaborative protocol and achievement of the target SBP, as well as the timing of withdrawal from intravenous antihypertensive drugs.

## 2. Materials and Methods

### 2.1. Ethical Considerations

This retrospective, single-center observational study was conducted in accordance with the STROBE (Strengthening the Reporting of Observational Studies in Epidemiology) reporting guidelines. The study protocol was approved by the Institutional Review Board of Kochi Medical School, Kochi University (approval number: 2023-101). The requirement for informed consent was waived owing to the use of anonymized data lacking personally identifiable information.

### 2.2. Patient Selection, Study Design, and Clinical Evaluation

**Figure 1:**
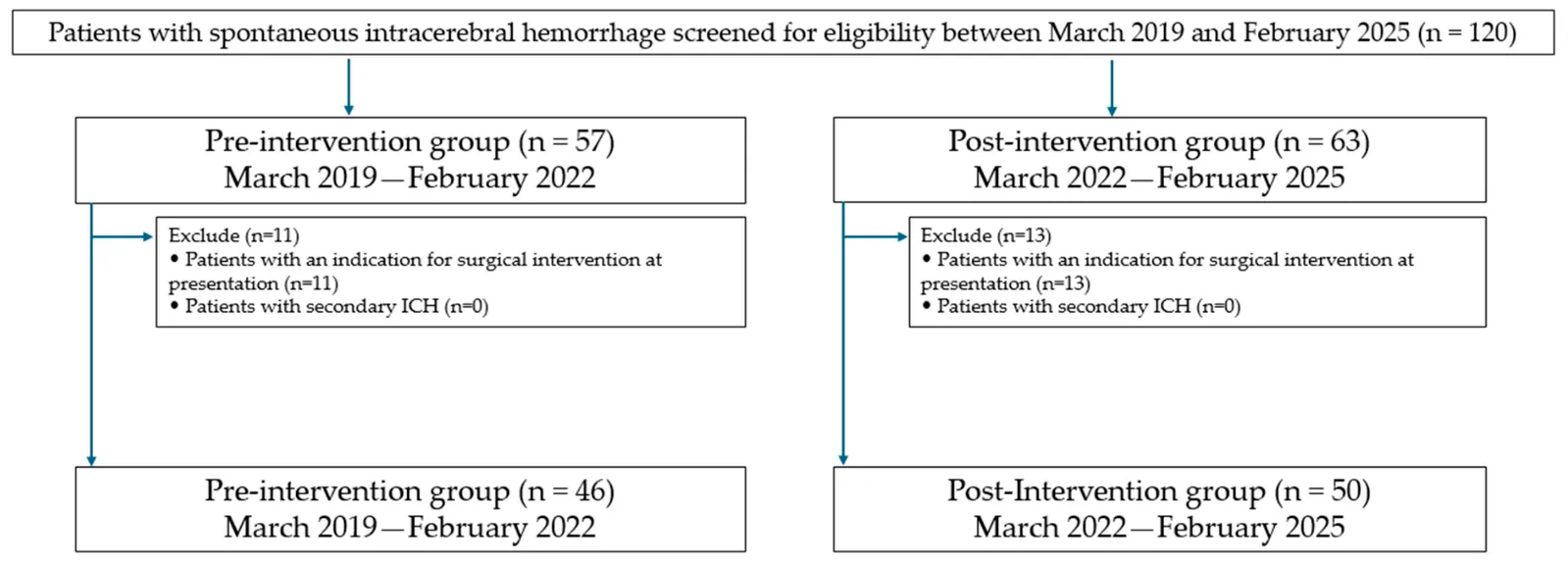
Flowchart of patient selection.

This study enrolled patients with primary ICH who were hospitalized for treatment at our institution between March 2019 and February 2025. The diagnosis of ICH was established using brain computed tomography (CT), and initial hematoma volume was estimated from CT images using the standardized ABC/2 method. Patients with ICH caused by vascular pathologies, including arteriovenous malformations, brain tumors, and trauma, were excluded. No patients with secondary ICH were identified among the screened patients. Patients who required surgical treatment for ICH at presentation were excluded. A total of 120 patients were screened for eligibility, including 57 patients during the pre-intervention period and 63 during the post-intervention period. Of these, 24 patients were excluded because of an indication for surgical intervention at presentation (11 in the pre-intervention period and 13 in the post-intervention period). No patients were subsequently excluded because of neurological deterioration requiring rescue surgery after admission. A total of 96 patients were enrolled in the final analysis (46 in the pre-intervention group and 50 in the post-intervention group), as illustrated in Figure 1. A formal sample size calculation was not performed because this retrospective study included all eligible patients treated during the predefined study period. This retrospective cohort study investigated the association between implementation of a novel protocol and blood pressure control and withdrawal from intravenous antihypertensive agents in patients with acute ICH by comparing patients managed pre-intervention (46 patients, March 2019–February 2022) and the post-intervention period (50 patients, March 2022–February 2025). The association between the novel protocol and achievement of target SBP during the first 7 days after onset, as well as the timing of withdrawal from intravenous antihypertensive agents, was evaluated. The primary outcome was achievement of the target SBP from 24 h after onset until day 7. The observation period was divided into six consecutive 24-h intervals (24–48, 48–72, 72–96, 96–120, 120–144, and 144–168 h after symptom onset). Target achievement was defined as a maximum recorded SBP < 140 mmHg in every interval. All 96 patients had recorded SBP measurements in each of the six intervals. No patients were discharged within seven days after ICH onset, and no in-hospital deaths occurred. Therefore, no observation intervals were missing for the assessment of the primary outcome. Patients with any recorded SBP ≥ 140 mmHg during these intervals were classified as not achieving the target. Symptom onset time was determined by the treating physician based on the available clinical information and documented in the electronic medical records. When the exact onset time was uncertain, the physician-documented onset time was used as the reference for defining the observation intervals. Secondary outcomes included time to withdrawal from intravenous nicardipine, hematoma expansion, constipation, delirium, duration of delirium, and renal function. Additional safety outcomes included early neurological deterioration, documented symptomatic hypotension, acute kidney injury, hyperkalemia requiring treatment, documented angioedema, and in-hospital mortality. Age, sex, body mass index, National Institutes of Health Stroke Scale score on admission, location of ICH, and use of oral antihypertensive drugs were retrieved from the electronic medical records as the baseline patient characteristics. Additional baseline characteristics included admission SBP, Glasgow Coma Scale score, initial hematoma volume, and the presence of intraventricular hemorrhage. Target SBP in each patient group was defined as SBP < 140 mmHg from 24 h after onset to day 7. The highest SBP and cumulative total nicardipine dose on each day were recorded to visualize daily trends. Because SBP measurements were obtained intermittently, target achievement was based on recorded measurements and did not establish continuous maintenance of SBP within the target range. The timing of nicardipine withdrawal was determined by the time of final discontinuation of the infusion, regardless of the presence of temporary discontinuation. Time to discontinuation was evaluated in hours. Hematoma expansion was defined as a >33% increase in hematoma volume compared with the baseline hematoma within 72 h after symptom onset [3,4]. Adverse events associated with continuous intravenous infusion, including constipation and delirium, and safety measures, such as renal function, were also evaluated. Constipation was defined as less than 3 defecations by day 7 [7]. The presence of delirium was clinically assessed by physicians using the Confusion Assessment Method (CAM) [8,9], and relevant clinical observations were documented in the medical and nursing records. In patients who developed delirium, the duration was defined as the number of days for which delirium was recorded by a ward nurse in the electronic medical records. Delirium duration was analyzed only among patients who developed delirium (11 in the pre-intervention group and 14 in the post-intervention group). Renal dysfunction associated with antihypertensive drugs was evaluated by laboratory testing of serum creatinine and the estimated glomerular filtration rate (eGFR) on admission and on day 5. Early neurological deterioration (END) was defined as an increase of ≥ 4 points in the National Institutes of Health Stroke Scale (NIHSS) score or a decrease of ≥ 2 points in the Glasgow Coma Scale (GCS) score from baseline within 24 h after ICH onset. The minimum recorded SBP during the first 24 h after ICH onset was defined as the lowest recorded SBP value during this period. Patients with at least one recorded SBP below 100 mmHg were identified. Documented symptomatic hypotension was assessed on the basis of clinical symptoms or findings associated with blood pressure reduction recorded in the medical records. Acute kidney injury (AKI) was evaluated retrospectively using available serum creatinine measurements and clinical documentation. AKI was retrospectively evaluated according to the serum creatinine criteria of the Kidney Disease: Improving Global Outcomes (KDIGO) 2012 guidelines [10]. AKI was defined as an increase in serum creatinine of ≥0.3 mg/dL within 48 h or an increase to ≥1.5 times the baseline value within 7 days. Available serum creatinine measurements and clinical documentation were reviewed to identify AKI. Urine output criteria were not applied because standardized urine output data were unavailable.

Hyperkalemia requiring treatment was assessed based on documented treatment for hyperkalemia. In-hospital mortality was defined as death occurring during the index hospitalization. An additional retrospective safety assessment was based on available clinical documentation and laboratory results. Because the frequency and timing of laboratory testing were not standardized, transient or unrecognized acute kidney injury could not be excluded.

A total of 120 patients with spontaneous intracerebral hemorrhage were screened for eligibility between March 2019 and February 2025. Of these, 57 patients were identified during the pre-intervention period (March 2019–February 2022) and 63 patients during the post-intervention period (March 2022–February 2025). A total of 24 patients were excluded because of an indication for surgical intervention at presentation (*n* = 24; pre-intervention, *n* = 11; post-intervention, *n* = 13). No patients with secondary intracerebral hemorrhage were identified within the screening cohort. The final analysis included 96 patients: 46 in the pre-intervention group and 50 in the post-intervention group.

### 2.3. Institutional Policy of Blood Pressure Control for ICH Before and After Introduction of the Protocol: What Has Changed?

In our facility, an institutional agreement on blood pressure control for ICH was available before March 2022. Although the target SBP was determined to be <140 mmHg for 7 days after onset, detailed dose control of intravenous antihypertensive drugs (nicardipine), choice of oral antihypertensive drugs, and timing of transition from intravenous to oral medication were left to the discretion of the attending physicians. In March 2022, pharmacists and physicians collaborated to establish a standardized protocol to achieve better blood pressure control and earlier withdrawal of intravenous antihypertensive drugs. The protocol simultaneously introduced a more intensive SBP target during the first 24 h, standardized nicardipine titration, and a predefined approach to oral antihypertensive treatment. This novel protocol is described hereafter, focusing on what has changed and what has remained unchanged before and after the introduction of the protocol. Pharmacists reviewed medication histories, evaluated antihypertensive therapy, assessed potential contraindications and adverse drug reactions, and proposed medication adjustments according to the institutional protocol. Physicians retained responsibility for prescribing decisions and treatment modifications, while nurses performed blood pressure monitoring and administered intravenous antihypertensive treatment according to clinical orders and institutional procedures. Pharmacists were authorized to recommend medication adjustments but did not independently prescribe medications or modify prescribed doses; final prescribing decisions and treatment modifications remained the responsibility of physicians. Because several treatment components were introduced simultaneously, the independent contribution of pharmacist involvement could not be determined.

#### 2.3.1. Daily Measurement of Blood Pressure (Unchanged)

Blood pressure was measured by ward nurses using an automatic monitor. The same model of automated blood pressure monitor was used throughout both the pre-intervention and post-intervention periods. During the initial phase, within 12 h of admission, measurements were taken at 5- to 15-min intervals. Subsequently, once blood pressure had stabilized, the intervals were extended to 30- to 60-min intervals. After 12 h of onset, measurements were taken at 4-h intervals. There were no changes in the institutional blood pressure measurement procedures before or after introduction of the protocol.

#### 2.3.2. Target SBP Within 24 h of Onset (Changed) and from 24 h to 7 Days (Unchanged)

Before the introduction of the novel protocol, blood pressure control within 24 h of onset targeted an SBP of <140 mmHg. After the introduction of the protocol, the target SBP within 24 h of onset was changed to 100 ≤ SBP < 120 mmHg. From 24 h to 7 days, the target SBP remained unchanged at <140 mmHg before and after introduction of the novel protocol.

The institutional blood pressure management protocol was developed in March 2022 with reference to evidence available at that time, including the INTERACT2 (2013) and ATACH-2 (2016) randomized controlled trials [3,4], the analysis by Toyoda et al. (2019) [11], and the individual participant data meta-analysis by Moullaali et al., published online in November 2021 [12]. These studies informed the development of the protocol, particularly its approach to early blood pressure reduction and subsequent blood pressure management in acute ICH. However, they did not directly establish the safety or efficacy of targeting SBP at 100 ≤ SBP < 120 mmHg during the first 24 h. Therefore, the specific SBP target and nicardipine titration procedures were determined as part of an institution-specific treatment strategy rather than being directly derived from established guideline recommendations.

The protocol was implemented in March 2022, before publication of the 2022 AHA/ASA and 2025 ESO/EANS guidelines, and the initial SBP target of 100 ≤ SBP < 120 mmHg was not established as a guideline-recommended target. In particular, contemporary guidelines caution against excessive acute blood pressure reduction, including SBP lowering below 130 mmHg in selected patients with mild-to-moderate ICH. Treatment decisions remained subject to the attending physician’s clinical judgment.

#### 2.3.3. Blood Pressure Control with Intravenous Antihypertensive Drugs (Changed)

Immediately following admission, the patient’s blood pressure was controlled using continuous intravenous infusion of nicardipine (1 mg/mL). Nicardipine was administered without dilution at a concentration of 1 mg/mL using a syringe pump through a peripheral intravenous line. Prior to the introduction of the novel protocol, dose control of nicardipine was left to the discretion of the attending physician or the on-call doctor. As such, increasing or decreasing the hourly infusion rate by 1 mL/h or 2 mL/h, and even rapid infusion of 1 mL–2 mL within a few seconds, was possible. Following the introduction of the novel protocol, the dose of nicardipine infusion was controlled according to the protocol outlined in Table 1. Nicardipine infusion was initiated at 2 mL/h (2 mg/h), adjusted in increments or decrements of 1 mL/h (1 mg/h), and limited to a maximum rate of 15 mL/h (15 mg/h). In brief, the hourly infusion rate of nicardipine was changed by only 1 mL/h to maintain SBP at 100 ≤ SBP < 120 mmHg within 24 h of onset and 120 ≤ SBP < 140 mmHg thereafter; rapid infusion was not allowed. In addition to the criteria for decreasing the nicardipine dose when the SBP was below 100 mmHg within 24 h of onset and below 120 mmHg thereafter, the nicardipine infusion was decreased by 1 mL/h when the target SBP was measured two consecutive times for withdrawal. Nicardipine infusion rates were reassessed and adjusted according to changes in SBP and the patient’s clinical condition. No fixed interval for reassessment of the infusion rate was specified in the institutional protocol. The maximum infusion rate of 15 mL/h (15 mg/h) was consistent with the maximum nicardipine infusion rate used in the ATACH-2 trial, although the starting dose, titration increments, and infusion preparation differed from those used in that study [4].

#### 2.3.4. Oral Antihypertensive Drugs (Changed)

Before the introduction of the novel protocol, oral amlodipine besilate was conventionally initiated for subacute and chronic blood pressure control; however, the dose and timing were chosen at the discretion of the attending physicians. In addition, the choice of a second oral antihypertensive drug when the first drug was insufficiently effective was not standardized. Following the introduction of the novel protocol, sacubitril/valsartan 200 mg once daily was determined to be the first oral antihypertensive drug and was generally initiated on the morning following admission. When nicardipine administration was still required 48 h after initiating sacubitril/valsartan, cilnidipine 10 mg once daily was considered as a second oral antihypertensive drug. Although the above-mentioned drugs were recommended, the choice of oral antihypertensive drugs was ultimately determined by the attending physician in accordance with the previous antihypertensive drugs administered before onset, patient preference, drug contraindications, and history of allergy. Before initiating sacubitril/valsartan, pharmacists reviewed patients’ medical histories, concomitant medications, potential contraindications and drug interactions, renal function, and serum potassium levels. Based on these assessments and the patient’s clinical condition, the attending physician determined whether sacubitril/valsartan was appropriate and made the final prescribing decision. The choice of sacubitril/valsartan was part of the institutional protocol and was not based on established evidence of superiority over conventional oral antihypertensive agents in acute ICH.

### 2.4. Statistical Analyses

Data distribution was assessed using the Shapiro–Wilk test, and non-parametric methods were employed when a normal distribution could not be assumed. The Mann–Whitney U and Fisher’s exact tests were used to compare continuous and categorical variables, respectively. Effect estimates and their 95% confidence intervals (CIs) were reported where appropriate. To examine the association between protocol implementation and achievement of the primary outcome while accounting for potential confounding, a multivariable logistic regression model was constructed. The dependent variable was achievement of the target SBP in all six consecutive 24-h intervals from 24 to 168 h after ICH onset. The independent variables were protocol implementation (post-intervention versus pre-intervention), baseline hematoma volume, admission SBP, admission Glasgow Coma Scale (GCS) score, and the presence of intraventricular hemorrhage (IVH). Adjusted odds ratios (aORs) and 95% CIs were calculated. All 96 patients were included in this analysis.

Interrupted time-series (ITS) analysis, which enables the consideration of background trends and periodicity, was conducted to evaluate the association between implementation of the blood pressure management protocol and the target SBP achievement rate from 24 h after onset until day 7 [13]. Although the study period comprised 72 months (36 months before and 36 months after protocol implementation), the ITS analysis was conducted using 12 consecutive six-month intervals, comprising six pre-intervention and six post-intervention intervals.

This aggregation was adopted because some individual months had no eligible patients, resulting in undefined monthly target achievement rates. The number of patients achieving the target SBP and the total number of eligible patients were calculated for each interval. Because the outcome was a proportion and the denominators varied across intervals, segmented regression was performed using a grouped binomial generalized linear model with a logit link, rather than ordinary least-squares regression of the achievement rates. The numbers of patients achieving and not achieving the target SBP were used as the binomial response, thereby accounting for differences in patient numbers across intervals. The interval-specific data are presented in Supplementary Table S2.

The independent variables included the time elapsed since study initiation, an indicator representing the intervention period (pre-intervention vs. post-intervention), and time elapsed post-intervention. Time was expressed in six-month units. March 2022 was classified as the beginning of the post-intervention period, and time after intervention was coded as 0 in the first post-intervention interval and increased by one for each subsequent interval. The model estimated the pre-intervention trend, immediate level change, and change in slope after intervention. Regression coefficients were reported on the log-odds scale, together with odds ratios and Wald 95% confidence intervals. Residual autocorrelation was assessed using the autocorrelation function of time-ordered Pearson residuals and Ljung–Box tests at lags 1 and 2. Overdispersion was evaluated using the Pearson chi-square statistic divided by the residual degrees of freedom. The primary model retained the binomial variance assumption, and no autocorrelation-specific adjustment was applied. Sensitivity analyses included a season-adjusted model with a binary indicator distinguishing March–August from September–February, profile-likelihood confidence intervals, Firth penalized logistic regression, and leave-one-interval-out analysis. Detailed regression estimates, model diagnostics, and sensitivity analyses are provided in Supplementary Tables S3 and S4.

For comparisons across days following ICH onset, a non-parametric two-way ANOVA using the Aligned Rank Transform (ART) was conducted, with “each day” and “protocol” treated as fixed factors [14]. This allowed evaluation of the main effects and interactions of each factor. The Mann–Whitney U test was applied for between-group comparisons at each time point.

Time to intravenous nicardipine discontinuation was additionally evaluated using Kaplan–Meier analysis, and the survival distributions were compared using the log-rank test. The event was defined as final discontinuation of intravenous nicardipine, and all 96 patients experienced this event without censoring. Cox proportional hazards regression was performed to estimate the hazard ratio for discontinuation, and the proportional hazards assumption was assessed using Schoenfeld residuals. Because this assumption was not satisfied, restricted mean survival time (RMST) analysis was also conducted to provide an additional comparison that did not rely on proportional hazards [15]. For the RMST analysis, both groups were compared over the same 239-h period. This time point was selected because the longest observed time to nicardipine discontinuation was 240 h in the pre-intervention group and 239 h in the post-intervention group.

Delirium duration was analyzed among patients who developed delirium. Because the data were discrete and contained numerous tied observations, the Mann–Whitney U test was additionally evaluated using an exact conditional permutation distribution. The Hodges–Lehmann estimator was calculated from all pairwise differences between groups, and the rank-biserial correlation was used as a standardized effect-size measure. Confidence intervals for the rank-biserial correlation were estimated using stratified bootstrap resampling.

Statistical analyses for data used in Table 2, Table 3 and Table 4 were conducted using the EZR software (version 1.68; Saitama Medical Center, Jichi Medical University, Saitama, Japan; https://www.jichi.ac.jp/saitama-sct/SaitamaHP.files/statmedEN.html, accessed on 1 July 2025). EZR is an enhanced version of R designed to add statistical functions frequently used in biostatistics [16]. The interrupted time-series analyses, including the reanalysis of the interrupted time-series data and sensitivity analyses, were performed using R software (version 4.4.1; R Foundation for Statistical Computing, Vienna, Austria). A nonparametric two-way ANOVA was performed using the ARTool package (version 0.11.2). The significance level was set at two-tailed *p* < 0.05.

Additional multivariable, survival, and effect-size analyses were performed using R. The significance level was set at two-tailed *p* < 0.05. Secondary and exploratory analyses were interpreted cautiously because no formal adjustment for multiple comparisons was applied. No formal statistical analysis plan specifying all comparisons was established before data analysis. Additional multivariable and interrupted time-series analyses conducted during manuscript revision were considered post hoc and exploratory. No formal adjustment for multiple comparisons was applied; therefore, findings from secondary and exploratory analyses were interpreted cautiously.

## 3. Results

### 3.1. Patient Characteristics and Oral Antihypertensive Drugs Given

The baseline characteristics of the patients who underwent antihypertensive therapy for acute ICH are summarized in Table 2. The distributions of age, sex, body mass index, neurological severity at admission, admission SBP, premorbid use of antithrombotic drugs, hemorrhage location, and renal function did not show statistically significant between-group differences. However, nonsignificant comparisons do not establish baseline equivalence. The median baseline hematoma volume was 9.5 mL [IQR, 3.5–17.6] in the pre-intervention group and 4.7 mL [IQR, 3.1–11.7] in the post-intervention group (*p* = 0.210). Intraventricular hemorrhage was observed in 6 patients (13%) and 4 patients (8%), respectively. The oral antihypertensive drugs administered following admission significantly shifted from calcium channel blockers/renin–angiotensin system inhibitors/diuretics in the pre-intervention group to sacubitril/valsartan in the post-intervention group after implementation of the novel protocol. In particular, sacubitril/valsartan use increased from 0% (0/46) to 96% (48/50), indicating a substantial change in the oral antihypertensive regimen following protocol implementation. In the remaining two post-intervention patients, alternative antihypertensive treatment was selected at the discretion of the attending physicians. The specific reasons for not initiating sacubitril/valsartan could not be reliably determined from the retrospective medical records.

**Table 2:** Baseline characteristics of patients who underwent antihypertensive therapy for acute intracerebral hemorrhage during the pre- and post-intervention periods of the novel protocol.

Variable	Pre-Intervention (*n* = 46)	Post-Intervention (*n* = 50)	*p* Value
Baseline characteristics			
Age (years)	75 [64–84]	75 [63–80]	0.52
Male sex	26 (57)	29 (58)	1.00
BMI (kg/m^2^)	22 [21–25]	24 [22–26]	0.19
NIHSS	10 [4–15]	7 [4–12]	0.32
SBP on admission (mmHg)	169 [156–187]	170 [153–192]	0.93
Use of antithrombotic drug	11 (24)	12 (24)	1.00
Early blood pressure and safety outcomes			
Admission GCS score	15 [15–15]	15 [15–15]	0.27
Admission GCS of 15	41 (89)	40 (80)	0.22
Intraventricular hemorrhage	6 (13)	4 (8)	0.51
Baseline hematoma volume (mL)	9.5 [3.5–17.6]	4.7 [3.1–11.7]	0.21
Minimum recorded SBP within 24 h (mmHg)	113 [109–122]	107.5 [104–117]	**0.035**
Minimum recorded SBP < 100 mmHg within 24 h	2 (4)	3 (6)	1.00
Early neurological deterioration within 24 h	2 (4)	0 (0)	0.23
Documented symptomatic hypotension	0 (0)	0 (0)	—
Documented acute kidney injury	0 (0)	0 (0)	—
Hyperkalemia requiring treatment	0 (0)	0 (0)	—
Angioedema	0 (0)	0 (0)	—
In-hospital mortality	0 (0)	0 (0)	—
Location of hemorrhage			0.97
Putamen	8 (17)	12 (24)	
Thalamus	14 (30)	13 (26)	
Lobar/Subcortical	11 (24)	10 (20)	
Other location	13 (28)	15 (30)	
Serum creatinine (mg/dL)	0.69 [0.52–0.97]	0.74 [0.64–1.00]	0.27
eGFR (mL/min/1.73 m^2^)	78 [59–91]	66 [51–90]	0.41
Oral antihypertensive drug after admission			
Calcium channel blocker	36 (78)	22 (44)	**0.001**
RAS inhibitor	14 (30)	1 (2)	**<0.001**
Beta-blocker	2 (4)	0 (0)	0.24
Diuretic	5 (10)	0 (0)	**0.02**
Sacubitril/valsartan	0 (0)	48 (96)	**<0.001**

Data are expressed as the median [IQR] or number of patients (%). Other locations included cerebellar, brainstem, and intraventricular hemorrhages. Statistically significant *p* values are indicated in boldface. Early neurological deterioration (END) was defined as an increase of ≥4 points in NIHSS or a decrease of ≥2 points in GCS from baseline within 24 h after ICH onset. The minimum recorded SBP was defined as the lowest documented SBP during the first 24 h after onset. Safety outcomes were assessed retrospectively using available clinical and laboratory records. The absence of documented AKI or symptomatic hypotension does not exclude unrecognized events. AKI, acute kidney injury; BMI, body mass index; END, early neurological deterioration; eGFR, estimated glomerular filtration rate; GCS, Glasgow Coma Scale; IQR, interquartile range; NIHSS, National Institutes of Health Stroke Scale; RAS, renin–angiotensin system; SBP, systolic blood pressure.

### 3.2. Target SBP Achievement, Withdrawal from Intravenous Antihypertensive Drugs, Hematoma Expansion, and Adverse Events

Target SBP achievement, withdrawal from intravenous nicardipine, daily dose of nicardipine after admission, hematoma expansion, and potential adverse events associated with intravenous and oral antihypertensive medications are summarized in Table 3. The proportion of patients who achieved the target SBP from 24 h after onset until day 7 significantly increased from 20% (9/46 patients) in the pre-intervention group to 54% (27/50 patients) in the post-intervention group (*p* = 0.001). In a multivariable logistic regression analysis adjusting for baseline hematoma volume, admission SBP, admission Glasgow Coma Scale score, and intraventricular hemorrhage, protocol implementation remained associated with target SBP achievement (adjusted odds ratio, 5.51; 95% CI, 2.09–14.51; *p* = 0.0006) (Table 5). In an exploratory subgroup analysis according to hematoma location, the 7-day SBP target was achieved in 7 of 21 patients with lobar ICH (33.3%) and 20 of 48 patients with deep ICH (41.7%). No statistically significant difference was observed between the groups (Fisher’s exact test, *p* = 0.598; odds ratio, 0.70; 95% CI, 0.24–2.05) (Supplementary Table S1).

**Figure 2:**
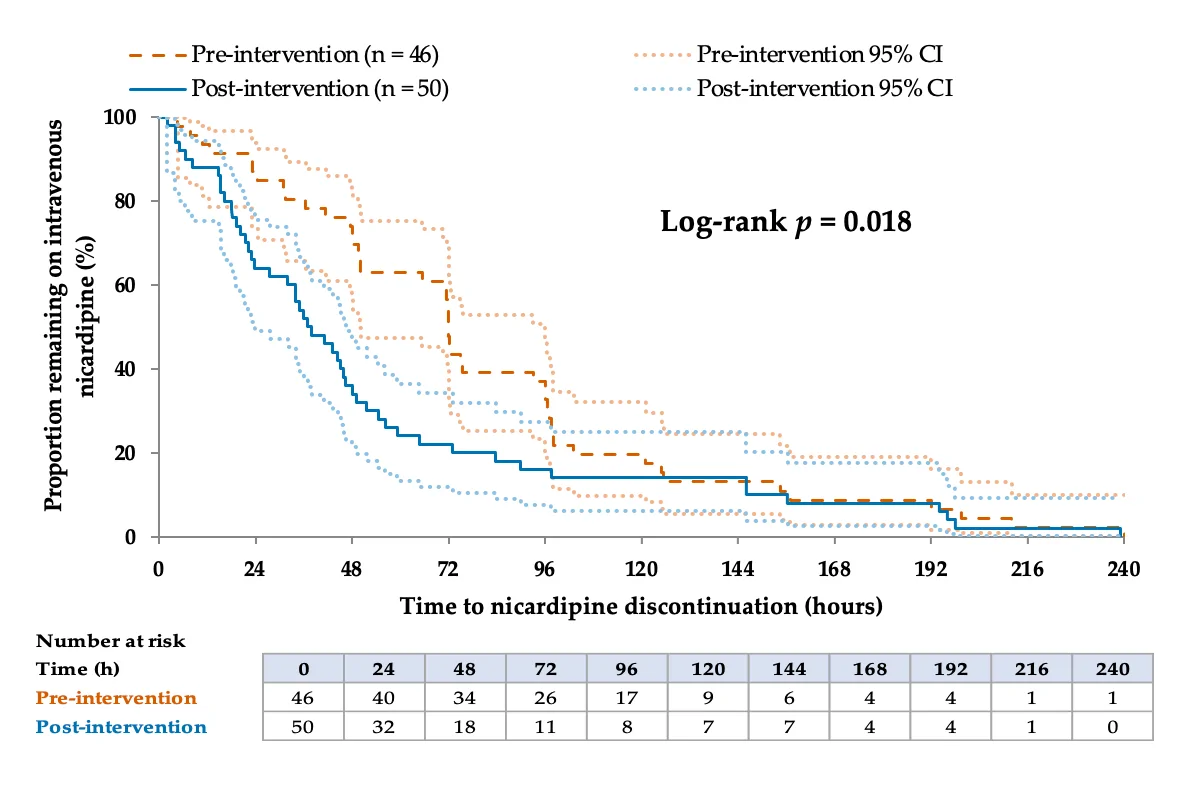
Kaplan–Meier curves for time to intravenous nicardipine discontinuation in the pre-intervention (*n* = 46) and post-intervention (*n* = 50) groups.

Time to intravenous nicardipine discontinuation was analyzed using the Kaplan–Meier method, and the results are presented in Section 3.3 and Figure 2. The daily dose of intravenous nicardipine in the post-intervention group was significantly larger at 0–24 h (74 [53–118] mg vs. 25 [19–50] mg, *p* < 0.001), not significantly different at 24–48 h (29 [8–81] vs. 48 [25–69], *p* = 0.54), and significantly smaller at 48–72 h (0 [0–28] vs. 25 [0–41], *p* = 0.01). A nonparametric two-way analysis of variance using the aligned rank transform demonstrated significant main effects of protocol implementation (F_1,94_ = 13.07, *p* < 0.001) and measurement day (F_7,658_ = 38.08, *p* < 0.001) on maximum SBP, without a significant protocol-by-day interaction (*p* = 0.106). Similarly, the cumulative total nicardipine dose showed significant main effects of protocol implementation (F_1,94_ = 8.03, *p* = 0.006) and measurement day (F_6,564_ = 165.28, *p* < 0.001), as well as a significant protocol-by-day interaction (F_6,564_ = 23.69, *p* < 0.001) (Table 4).

The incidence of hematoma expansion was not significantly different between the pre-intervention (5/46 patients, 10.9%) and post-intervention (3/50 patients, 6.0%) groups (risk ratio for post-intervention versus pre-intervention, 0.55; 95% CI, 0.14–2.18; Fisher’s exact test, *p* = 0.47).

No in-hospital deaths occurred in either group (0/46 and 0/50, respectively); consequently, there were no neurological or non-neurological deaths during hospitalization. Early neurological deterioration was documented in two patients (4.3%) in the pre-intervention group and none in the post-intervention group. No cases of documented symptomatic hypotension, acute kidney injury, hyperkalemia requiring treatment, or angioedema were identified in either group. During the first 24 h, the minimum recorded SBP was 113 mmHg [IQR, 109–122] in the pre-intervention group and 107.5 mmHg [IQR, 104–117] in the post-intervention group. A minimum recorded SBP below 100 mmHg occurred in 2 patients (4.3%) and 3 patients (6.0%), respectively.

**Table 3:** Achievement of target SBP from 24 h after onset until day 7, daily dose and withdrawal from intravenous nicardipine, hematoma expansion, and potential adverse events associated with intravenous and oral antihypertensive medication.

Outcome	Pre-Intervention (*n* = 46)	Post-Intervention (*n* = 50)	*p* Value
Target SBP achievement	9 (20)	27 (54)	**0.001**
Time to nicardipine discontinuation, Kaplan–Meier median (95% CI), h	72.5 [50.1–96.0]	37.5 [23.8–46.5]	**0.018**
Daily nicardipine dose (mg)			
0–24 h	25 [19–50]	74 [53–118]	**<0.001**
24–48 h	48 [25–69]	29 [8–81]	0.54
48–72 h	25 [0–41]	0 [0–28]	**0.01**
Hematoma expansion	5 (11)	3 (6)	0.47
Constipation	15 (33)	16 (32)	1.00
Delirium	11 (24)	14 (28)	0.82
Duration of delirium (days)	2 [2,3]	1 [1,2]	**0.001**
Serum creatinine (mg/dL) day 5	0.74 [0.57–0.99]	0.70 [0.59–0.86]	0.45
eGFR (mL/min/1.73 m^2^) day 5	71 [59–84]	80 [61–88]	0.22

Data are expressed as the median [IQR] or number of patients (%), unless otherwise specified. Statistically significant *p* values are indicated in boldface. Time to intravenous nicardipine discontinuation was analyzed using the Kaplan–Meier method, and the between-group difference was assessed using the log-rank test. For this outcome, values are presented as Kaplan–Meier estimated medians (95% confidence intervals). Delirium duration was analyzed only among the 25 patients who developed delirium (11 in the pre-intervention group and 14 in the post-intervention group); patients who did not develop delirium were excluded from this analysis. Abbreviations: BP, blood pressure; eGFR, estimated glomerular filtration rate; IQR, interquartile range.

**Table 4:** Temporal changes in blood pressure control and nicardipine use before and after protocol implementation.

Outcome	Pre-Intervention	Post-Intervention	*p* Value
Maximum SBP (mmHg)			
SBP on admission (mmHg), median [IQR]	169 [156–187]	170 [153–192]	0.93
SBP on 0–24 h (mmHg), median [IQR]	147 [139–154]	140 [129–145]	0.002
SBP on 24–48 h (mmHg), median [IQR]	146 [136–157]	135 [128–140]	<0.001
SBP on 48–72 h (mmHg), median [IQR]	144 [133–158]	138 [131–149]	0.096
SBP on 72–96 h (mmHg), median [IQR]	148 [131–156]	137 [127–148]	0.005
SBP on 96–120 h (mmHg), median [IQR]	151 [135–158]	139 [131–150]	0.029
SBP on 120–144 h (mmHg), median [IQR]	147 [136–160]	138 [126–148]	0.001
SBP on 144–168 h (mmHg), median [IQR]	144 [134–162]	139 [130–147]	0.047
Total Nicardipine Dose (mg)			
0–24 h (mg), median [IQR]	25 [19–50]	74 [53–118]	<0.001
24–48 h (mg), median [IQR]	48 [25–69]	29 [8–81]	0.54
48–72 h (mg), median [IQR]	25 [0–41]	0 [0–28]	0.01
72–96 h (mg), median [IQR]	0 [0–25]	0 [0–0]	0.008
96–120 h (mg), median [IQR]	0 [0–0]	0 [0–0]	0.21
120–144 h (mg) median [IQR]	0 [0–0]	0 [0–0]	0.68
144–168 h (mg), median [IQR]	0 [0–0]	0 [0–0]	0.35

Data are presented as median [interquartile range]. *p* values represent between-group comparisons at each time point using the Mann–Whitney U test. Abbreviations: SBP, systolic blood pressure; IQR, interquartile range.

The incidence of constipation and renal function associated with acute antihypertensive treatment were comparable between the pre- and post-intervention groups.

The incidence of delirium was 23.9% (11/46) in the pre-intervention group and 28.0% (14/50) in the post-intervention group (*p* = 0.82). Among patients who developed delirium (*n* = 25), the median duration was 2 days [IQR, 2–3] and 1 day [IQR, 1–2], respectively. The Hodges–Lehmann estimate of the between-group difference (pre-intervention minus post-intervention) was 1.0 day, and the rank-biserial correlation was 0.62 (95% BCa bootstrap CI, 0.17–0.85).

**Table 5:** Multivariable logistic regression analysis of 7-day target SBP achievement.

Variable	Adjusted OR	95% CI (Wald)	*p* Value	95% CI (Profile Likelihood)
Protocol implementation	5.51	2.09–14.51	0.0006	2.17–15.33
Baseline hematoma volume (mL)	1.003	0.958–1.051	0.892	0.957–1.052
Admission SBP (mmHg)	0.984	0.965–1.003	0.100	0.964–1.003
Admission GCS	0.838	0.602–1.165	0.293	0.592–1.174
Intraventricular hemorrhage	2.20	0.47–10.29	0.316	0.46–10.67

Multivariable logistic regression analysis included all 96 patients, of whom 36 achieved the 7-day SBP target. The model included protocol implementation, baseline hematoma volume, admission SBP, admission GCS score, and IVH. Adjusted odds ratios (ORs) and 95% confidence intervals (CIs) were calculated. Wald 95% CIs and *p* values are reported, with profile-likelihood CIs provided for comparison. CI, confidence interval; GCS, Glasgow Coma Scale; IVH, intraventricular hemorrhage; OR, odds ratio; SBP, systolic blood pressure.

The orange dashed line represents the pre-intervention group, and the blue solid line represents the post-intervention group. Dotted lines indicate the corresponding 95% confidence intervals. The vertical axis shows the proportion of patients remaining on intravenous nicardipine, and the horizontal axis shows time to discontinuation in hours. The number of patients at risk at each time point is presented below the graph. All 96 patients experienced the discontinuation event, and no observations were censored. The log-rank test indicated a significant difference between groups (*p* = 0.018).

### 3.3. Kaplan–Meier Curves for Time to Nicardipine Discontinuation

Kaplan–Meier analysis was performed to compare the time to intravenous nicardipine discontinuation between the pre-intervention and post-intervention groups. All 96 patients experienced the discontinuation event, and no observations were censored. The Kaplan–Meier curves indicated earlier discontinuation in the post-intervention group (log-rank *p* = 0.018). The estimated median time to discontinuation was 72.5 h (95% CI, 50.1–96.0) in the pre-intervention group and 37.5 h (95% CI, 23.8–46.5) in the post-intervention group. Cox proportional hazards regression yielded a hazard ratio of 1.62 (95% CI, 1.08–2.44; *p* = 0.020) for nicardipine discontinuation in the post-intervention group compared with the pre-intervention group. However, the proportional hazards assumption was not satisfied (Schoenfeld residual test, *p* = 0.013). Therefore, restricted mean survival time (RMST) analysis was additionally performed. At a restriction time of 239 h, the RMST was 81.1 h in the pre-intervention group and 56.0 h in the post-intervention group, corresponding to a between-group difference of 25.1 h (95% CI, 3.0–47.2; *p* = 0.026). These findings indicated an association between protocol implementation and earlier intravenous nicardipine discontinuation (Figure 2).

### 3.4. Evaluation of the Protocol with ITS Analyses

**Figure 3:**
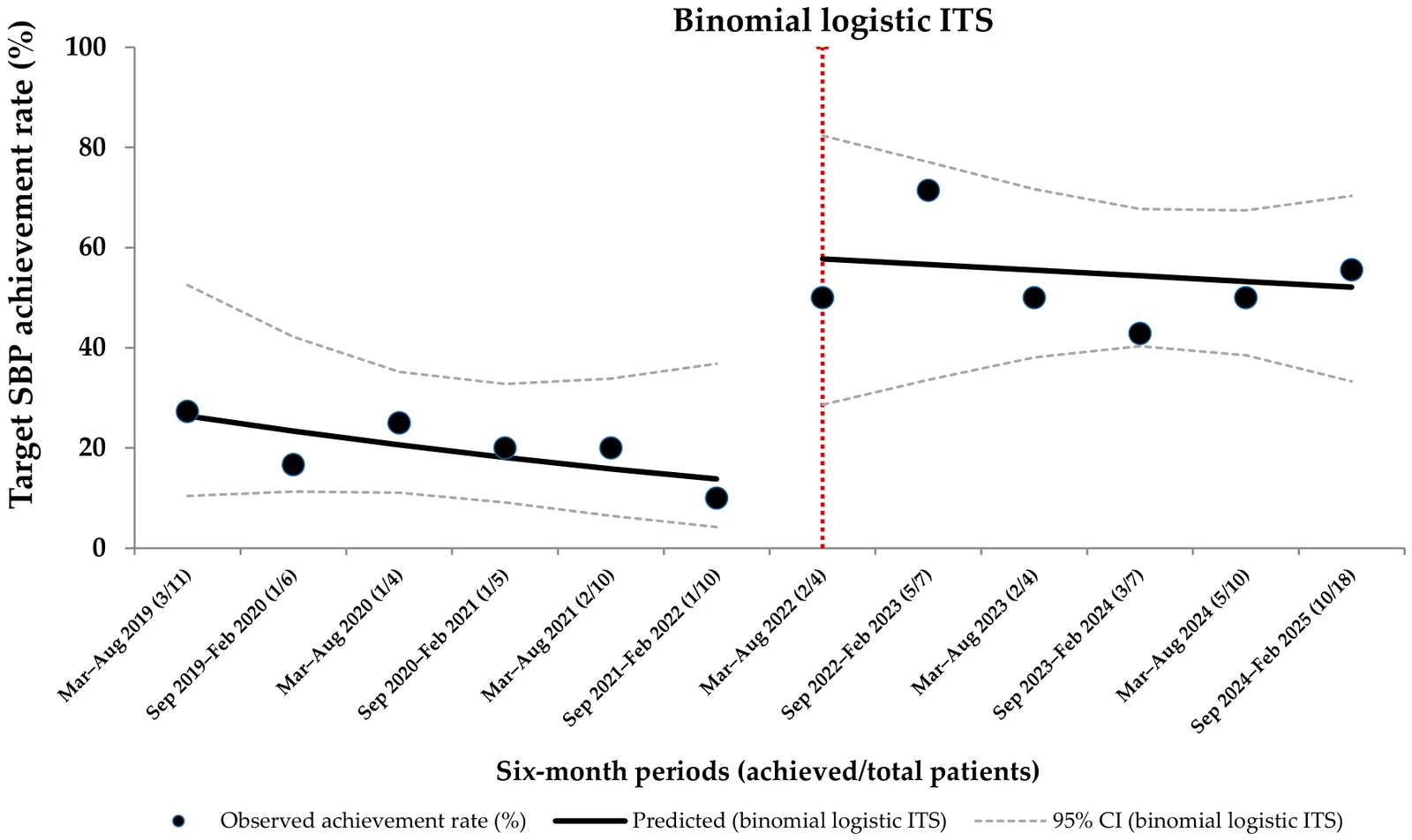
Interrupted time-series analysis of 7-day target SBP achievement following implementation of the pharmacist–physician collaborative protocol. Black dots connected by gray lines represent observed target SBP achievement rates in each of 12 consecutive six-month intervals. Dark solid lines represent fitted probabilities estimated using segmented binomial logistic regression, and light gray lines indicate the corresponding 95% confidence intervals. The vertical red line indicates protocol implementation in March 2022. The numbers in parentheses along the horizontal axis indicate the number of patients achieving the target SBP and the total number of eligible patients in each interval (achieved/total). Abbreviations: ITS, interrupted time series; SBP, systolic blood pressure.

To evaluate the association between implementation of the protocol and target SBP achievement while accounting for underlying temporal trends, ITS analyses were performed. The 72-month study period was divided into 12 consecutive six-month intervals, comprising six pre-intervention and six post-intervention intervals. Because the denominators varied across intervals, a segmented grouped binomial logistic regression model was used to analyze the number of patients achieving the target SBP relative to the number of eligible patients in each interval. The analysis demonstrated a significant immediate level change following protocol implementation (odds ratio [OR], 10.03; 95% CI, 1.31–76.78; *p* = 0.026) (Figure 3). The pre-intervention temporal trend was not statistically significant (OR per six-month interval, 0.85; 95% CI, 0.58–1.25; *p* = 0.410). The change in temporal slope after implementation was also not statistically significant (OR, 1.12; 95% CI, 0.68–1.86; *p* = 0.654). In a sensitivity analysis adjusting for seasonality by distinguishing March–August from September–February, the immediate level change remained statistically significant (OR, 10.21; 95% CI, 1.28–81.48; *p* = 0.028) (Supplementary Figure S1). These ITS findings were exploratory because the analysis included only 12 six-month intervals with relatively small and unequal numbers of patients, and potential time-varying confounding could not be excluded. The corresponding regression coefficients and detailed model estimates are provided in Supplementary Table S3. The immediate level-change coefficient was β = 2.306 in the primary model and β = 2.323 in the season-adjusted model. No evidence of overdispersion was observed (Pearson χ^2^/residual degrees of freedom = 0.218). The lag-1 autocorrelation coefficient of the Pearson residuals was −0.172, and Ljung–Box tests were not statistically significant at lag 1 (*p* = 0.502) or lag 2 (*p* = 0.073). However, the limited number of intervals reduced the power to detect residual autocorrelation. Detailed diagnostic results and sensitivity analyses are presented in Supplementary Table S4.

## 4. Discussion

Overall, the present study demonstrated that implementation of the blood pressure management protocol developed collaboratively by pharmacists and physicians was associated with a higher rate of target SBP achievement rates within 7 days of ICH onset. In addition, time to intravenous nicardipine discontinuation was shorter in the post-intervention group. Among patients who developed delirium, a shorter duration was also observed. However, these findings represent associations with protocol implementation and do not establish improvements in clinical outcomes or the independent effectiveness of pharmacist involvement.

ICH is a major contributor to high mortality and morbidity. Hematoma expansion, which may be associated with elevated blood pressure during the early phase of ICH, is strongly associated with poor functional outcomes; as such, appropriate blood pressure management to limit hematoma expansion is crucial in acute-phase treatment [1,3,14]. Although the target SBP has been extensively investigated and advocated in several clinical trials and guidelines [1,3,14], antihypertensive treatment strategies to achieve the target SBP in the acute and subacute phases have not been well-defined [3,12,17,18]. Current guidelines support careful and sustained blood pressure reduction toward approximately 140 mmHg in selected patients with mild-to-moderate ICH, while cautioning against excessive acute reduction, particularly below 130 mmHg. While intravenous antihypertensive agents, including calcium channel blockers, are recommended to rapidly lower blood pressure in the acute phase, their titration and subsequent transition to oral treatment may vary according to institutional practice and clinical circumstances. In addition, although intravenous antihypertensive agents are generally transitioned to oral drugs when clinically appropriate, the choice and dose of oral drugs or timing of withdrawal from intravenous agents have not been well defined, resulting in the prolonged use of intravenous antihypertensive agents and failure to facilitate patient activity [2,4,5,6]. As such, a treatment protocol that facilitates consistent blood pressure management and appropriate transition from intravenous to oral antihypertensive therapy is anticipated.

In the present study, the target SBP in the subacute phase (<140 mmHg, from 24 h to 7 days) was more frequently achieved following implementation of the novel protocol. This association remained after adjustment for baseline hematoma volume, admission SBP, admission Glasgow Coma Scale score, and intraventricular hemorrhage (adjusted odds ratio, 5.51; 95% CI, 2.09–14.51). Nevertheless, residual confounding cannot be excluded. There are several possible reasons for these results. First, the target SBP was 100 ≤ SBP < 120 mmHg during the initial 24 h under the novel protocol, while SBP < 140 mmHg was targeted before protocol implementation. The novel protocol was developed partly on the basis of previous Japanese individual-participant data suggesting an association between achieved SBP and subsequent clinical outcomes [11]. However, the specific SBP target of 100 ≤ SBP < 120 mmHg was determined as part of an institution-specific treatment strategy.

The lower SBP target during the initial 24 h may have facilitated achievement of SBP < 140 mmHg during the subsequent observation period. However, the 2023 analysis by Toyoda et al. [19] evaluated achieved SBP rather than the effects of randomization to a target of 100–120 mmHg. Moreover, it excluded patients with infratentorial hemorrhage, whereas the present study included patients with cerebellar and brainstem hemorrhage. Therefore, its findings cannot be directly generalized to the entire population in our study.

Importantly, the institutional target of 100 ≤ SBP < 120 mmHg during the first 24 h was more intensive than the target generally recommended by the 2022 AHA/ASA guideline, which cautions that acute SBP lowering below 130 mmHg may be harmful in patients with mild-to-moderate ICH [17]. Similarly, the 2025 European Stroke Organisation/European Association of Neurosurgical Societies (ESO/EANS) guideline supports early SBP reduction below 140 mmHg in selected patients with minor-to-moderate ICH and emphasizes appropriate blood pressure management during the subsequent seven days [20]. These recommendations highlight the importance of individualized blood pressure management according to hemorrhage severity and clinical condition [20]. Accordingly, the higher rate of subsequent SBP target achievement in our study should not be interpreted as evidence of the safety or clinical superiority of this intensive early target. Second, the novel protocol defined the first oral antihypertensive drug as sacubitril/valsartan 200 mg/day generally administered on the morning following admission, while amlodipine, a calcium channel blocker, was conventionally started at an arbitrary timing. The choice of sacubitril/valsartan in the novel protocol depended on our institutional preliminary experience of the substantial blood pressure-lowering effect of additional sacubitril/valsartan in non-stroke patients with intractable hypertension in whom initial amlodipine was ineffective. Sacubitril and valsartan decrease blood pressure through enhancement of natriuretic peptide activity via neprilysin inhibition and suppression of the renin–angiotensin–aldosterone system via angiotensin II type 1 receptor blockade, respectively [21,22]. Compared with calcium channel blockers, sacubitril/valsartan has been reported to provide sustained antihypertensive effects, suppress diurnal blood pressure fluctuations, and suppress blood pressure surges early in the morning [21,22]. We hypothesize that the addition of sacubitril/valsartan to intravenous nicardipine may have provided additional blood pressure-lowering effects through complementary pharmacological pathways [23]. However, these potential additive or synergistic effects were not directly evaluated in the present study. The superiority of sacubitril/valsartan over conventional oral antihypertensive therapy in patients with acute ICH therefore cannot be inferred from these findings.

In the present study, withdrawal from intravenous nicardipine occurred significantly earlier in the post-intervention group than in the pre-intervention group. Kaplan–Meier analysis showed an earlier time to discontinuation, and restricted mean survival time analysis provided additional support for this association. Sacubitril/valsartan may have stabilized blood pressure through the aforementioned multiple mechanisms in the subacute phase, potentially contributing to earlier withdrawal from intravenous nicardipine. However, the independent contribution of sacubitril/valsartan could not be determined because multiple components of blood pressure management were modified simultaneously. In addition, cilnidipine, a calcium channel blocker, was considered as the second oral antihypertensive drug in the novel protocol when nicardipine was still required 48 h after the first administration of sacubitril/valsartan. During the tapering of intravenous nicardipine, oral cilnidipine may have provided additional blood pressure-lowering effects in combination with sacubitril/valsartan, potentially facilitating earlier nicardipine withdrawal. However, this potential additive effect was not directly evaluated in the present study. Prolonged intravenous therapy may increase exposure to vascular access devices and potentially restrict patient mobility. Previous studies have highlighted the risks associated with intravascular devices and the importance of early mobilization in critically ill patients [24,25,26,27]. However, these complications were not comprehensively assessed as outcomes attributable to the duration of nicardipine infusion in this study. Delirium is commonly observed in patients with ICH and is associated with poorer functional outcomes and longer hospital stays [8,28]. Although the incidence of delirium did not differ significantly between groups, its duration was shorter among patients who developed delirium in the post-intervention group. This exploratory finding raises the possibility of a relationship with earlier discontinuation of intravenous nicardipine, which warrants further investigation. A causal relationship between nicardipine discontinuation and delirium duration cannot be established from the present data.

Prior studies have consistently demonstrated that standardized multidisciplinary protocols in acute critical care settings, including for sepsis, myocardial infarction, and mechanical ventilation in the intensive care unit, can improve treatment processes and outcomes [29,30,31,32,33,34]. Similarly, implementation of our pharmacist–physician collaborative protocol was associated with improved target SBP achievement and earlier withdrawal from intravenous nicardipine. These findings may reflect the combined effects of standardized antihypertensive titration, changes in oral antihypertensive therapy, medication monitoring, and facilitation of timely transition from intravenous to oral therapy by pharmacists and physicians. In addition to these process-related contributions, the pharmacological advantages of sacubitril/valsartan may also have contributed to the improved blood pressure control observed in the present study. However, the present study could not distinguish the effects of the overall protocol from those of individual antihypertensive agents. More specifically, although the choice of sacubitril/valsartan in the novel protocol was initially proposed by physicians based on their preliminary clinical experience, its potential pharmacological advantages, including possible additive effects when combined with calcium channel blockers, were considered by pharmacologists based on available evidence [21]. The choice of the second oral antihypertensive drug was also influenced by information provided by pharmacologists, which suggested that cilnidipine may offer a more favorable tolerability profile than amlodipine, although direct evidence regarding the prevention of angioedema associated with sacubitril/valsartan is lacking [35,36]. In addition, adverse events, including constipation and delirium, were carefully monitored by pharmacists in collaboration with physicians to support timely clinical assessment and intervention. Nevertheless, because protocol implementation involved simultaneous changes in SBP targets, nicardipine titration, and oral antihypertensive therapy, the independent effect of pharmacist involvement could not be determined.

The clinical characteristics and prognosis of ICH may differ according to hematoma location and underlying etiology. Previous studies have reported that lobar ICH has a distinct clinical profile and a more severe early prognosis than deep ICH, with non-hypertensive mechanisms, such as cerebral amyloid angiopathy, being particularly relevant in lobar hemorrhage [37]. In our exploratory subgroup analysis, the 7-day SBP target was achieved in 7 of 21 patients with lobar ICH (33.3%) and 20 of 48 patients with deep ICH (41.7%), without a statistically significant difference between the groups. However, this analysis was limited by the small number of patients and did not establish the absence of an association between hematoma location and target SBP achievement. Furthermore, small intracerebral hemorrhages may present with lacunar syndromes, sometimes referred to as hemorrhagic lacunar stroke [38]. Arboix et al. reported that this subgroup accounted for 7.4% of intracerebral hemorrhages in their hospital-based registry and was associated with more favorable early outcomes, including no in-hospital deaths [39]. Although baseline hematoma volume and neurological severity were assessed in the present study, hemorrhagic lacunar stroke was not specifically classified. Thus, its potential influence on target SBP achievement and clinical outcomes remains uncertain.

### Limitations

The present study has some limitations. First, this single-center retrospective study was susceptible to the influence of institutional policies, and prospective multicenter studies are necessary to generalize the results. Second, as this study employed a before-and-after design, rather than a randomized one, the observed effects may have been influenced by temporal changes in clinical practice, including evolving ICH management during the six-year study period, staff experience, or other unmeasured confounding factors. Although multivariable logistic regression was performed to adjust for baseline hematoma volume, admission SBP, admission Glasgow Coma Scale score, and intraventricular hemorrhage, residual confounding could not be excluded. Furthermore, because multiple changes in blood pressure management were introduced simultaneously, the independent contributions of pharmacist involvement and individual antihypertensive agents could not be determined. Although sacubitril/valsartan was administered to 96% of patients in the post-intervention group, comprehensive patient-level data on adherence to all protocol components, physician-directed deviations, and actual oral antihypertensive initiation times were unavailable. Therefore, we could not assess how variations in protocol implementation and treatment timing may have influenced the observed outcomes. Furthermore, the frequency and detailed nature of individual pharmacist interventions were not systematically recorded, preventing quantitative assessment of pharmacist intervention frequency and its independent contribution to the observed outcomes. Third, the safety of the intensive SBP target of 100 ≤ SBP < 120 mmHg during the first 24 h could not be adequately established in this retrospective study. Although no documented cases of symptomatic hypotension, acute kidney injury, hyperkalemia requiring treatment, or in-hospital mortality were identified in either group, the limited sample size and retrospective assessment may have resulted in unrecognized adverse events. In particular, serum creatinine measurements were not obtained at standardized intervals, and transient acute kidney injury could not be excluded. Furthermore, renal function was evaluated over a limited observation period, and the long-term safety of this intensive blood pressure management strategy remains uncertain, particularly in patients with impaired renal function at admission. Although protocol implementation was associated with improved target SBP achievement and earlier withdrawal from intravenous nicardipine, further studies are warranted to determine whether these improvements translate into better patient-centered clinical outcomes. Patient-centered outcomes, including hospital stay, functional status, and rehabilitation needs, were not comprehensively evaluated. Furthermore, the primary outcome was based on the maximum recorded SBP in each of six consecutive 24-h intervals rather than continuous blood pressure monitoring. Because target achievement was defined solely by an upper SBP threshold, the endpoint did not account for potentially excessive blood pressure reduction. Consequently, target achievement did not necessarily indicate sustained control throughout each interval. The interrupted time-series analysis was limited to 12 six-month intervals, with relatively small and unequal numbers of patients. The limited number of intervals may have reduced the reliability of estimates of temporal trends, seasonality, and autocorrelation. Although no statistically significant residual autocorrelation was detected, the small number of intervals limited the power of the diagnostic tests, and residual serial dependence could not be excluded. Furthermore, aggregation into six-month intervals may have obscured short-term temporal changes. The estimated immediate level change had a wide confidence interval, and statistical significance was not maintained in all leave-one-interval-out analyses. Therefore, the ITS findings should be interpreted cautiously and do not establish a causal effect of protocol implementation. In the analysis of time to nicardipine discontinuation, the proportional hazards assumption was not satisfied; restricted mean survival time analysis was therefore used as an additional measure. Although the same model of automated blood pressure monitor was used throughout the study period, retrospective data collection and uncertainty in the timing of symptom onset may have affected the classification of blood pressure measurements into the predefined intervals. Fourth, the premorbid control of hypertension before ictus may differ among patients with ICH; blood pressure was optimally controlled with multiple drugs in some patients, while it was uncontrolled in others, which may affect sensitivity to and interaction with drugs introduced after admission. Furthermore, premorbid functional status and precise onset-to-treatment intervals could not be reliably evaluated for all patients. Hemorrhagic lacunar stroke was not specifically classified, and subgroup comparisons according to hematoma location were exploratory. Finally, the sample size was relatively small, particularly for hematoma expansion, delirium, and safety outcomes, which may have limited the statistical power to detect differences between groups. Specifically, only eight patients experienced hematoma expansion, and the study was not adequately powered to determine whether the protocol reduced hematoma expansion. No in-hospital deaths occurred, preventing meaningful comparisons of mortality. Additionally, delirium duration was evaluated only among patients who developed delirium, limiting the interpretation of this secondary finding. Furthermore, delirium assessment frequency, clinical documentation practices, baseline cognitive status, sedative exposure, and observation duration were not fully characterized, and delirium-free days were not evaluated across the entire cohort.

Although blood pressure was generally measured at four-hour intervals, the exact number of measurements per patient could not be reliably determined from the available retrospective records. Therefore, potential differences in measurement frequency between patients or groups could not be assessed, which may have influenced the detection of transient SBP elevations and the classification of target achievement. Multiple exploratory comparisons were conducted without formal adjustment for multiplicity. Future prospective, multicenter studies with larger sample sizes are warranted to evaluate the safety and clinical outcomes of this standardized blood pressure management protocol. Further studies should also clarify the independent contributions of pharmacist involvement and changes in antihypertensive treatment strategies to the observed outcomes.

## 5. Conclusions

Overall, the present study found that implementation of our novel pharmacist–physician collaborative protocol for ICH, which was characterized by more intensive blood pressure control by intravenous nicardipine during the initial 24 h and predefined administration of oral antihypertensive drugs, was associated with a higher rate of target SBP achievement from 24 h to 7 days after onset. In addition, the timing of intravenous nicardipine withdrawal was significantly shorter in patients treated using the novel protocol. These findings suggest that implementation of our pharmacist–physician collaborative protocol was associated with improved target SBP achievement and earlier discontinuation of intravenous nicardipine in patients with ICH. However, further prospective studies are needed to establish the clinical effectiveness and safety of the intensive early SBP target and to determine the independent contributions of multidisciplinary collaboration and antihypertensive drug selection.

## Figures and Tables

**Table 1:** Overview of the pharmacist–physician collaborative standardized blood pressure management protocol for patients with intracerebral hemorrhage introduced in March 2022. Nicardipine was administered without dilution at a concentration of 1 mg/mL using a syringe pump. Therefore, 1 mL/h corresponded to 1 mg/h. Infusion adjustments were based on blood pressure measurements and clinical assessment without a fixed reassessment interval. Oral antihypertensive treatment could be individualized according to the attending physician’s judgment.

	Target SBP	Antihypertensive Agent for Blood Pressure Control
Within 24 h of onset	100 ≤ SBP < 120 mmHg	Nicardipine control: • Start nicardipine (1 mg/mL) at 2 mL/h (2 mg/h) when SBP ≥ 120 mmHg • Increase nicardipine by 1 mL/h (1 mg/h) when SBP remains ≥120 mmHg • Decrease nicardipine by 1 mL/h (1 mg/h) when SBP < 100 mmHg • Decrease nicardipine by 1 mL/h (1 mg/h) when SBP < 120 mmHg two consecutive times • Maximum dose: 15 mL/h (15 mg/h) • Start sacubitril/valsartan 200 mg once daily on the next morning of admission
From 24 h until day 7	120 ≤ SBP < 140 mmHg	Nicardipine control: • Increase nicardipine by 1 mL/h (1 mg/h) when SBP ≥ 140 mmHg • Decrease nicardipine by 1 mL/h (1 mg/h) when SBP < 120 mmHg • Decrease nicardipine by 1 mL/h (1 mg/h) when SBP < 140 mmHg two consecutive times • Maximum dose: 15 mL/h (15 mg/h) • Start cilnidipine 10 mg once daily when nicardipine administration was still required for 48 h after initiating sacubitril/valsartan

## Data Availability

The raw data supporting the conclusions of this article will be made available by the authors on request.

## Supplementary Materials

The following supporting information can be downloaded at: https://www.mdpi.com/article/10.3390/jcm15218733/s1, Figure S1: Season-adjusted ITS analysis of 7-day target SBP achievement following implementation of the pharmacist–physician collaborative protocol; Table S1: Seven-day SBP target achievement according to hematoma location. Table S2: Interval-specific patient counts and SBP target achievement rates used in the interrupted time-series analysis. Table S3: Regression coefficients and effect estimates from segmented binomial logistic regression. Table S4: Model diagnostics and sensitivity analyses of the interrupted time-series analysis.
