# Supplementary material for: Effects of a Pharmacist–Physician Collaborative Protocol for Acute Blood Pressure Management of Patients with Intracerebral Hemorrhage
Source: J Clin Med. 2026 Nov 9;15(21):8733. doi: 10.3390/jcm15218733 (PMC13763761; doi:10.3390/jcm15218733)
Supplement: Supplementary file 1 [file jcm-15-08733-s001.zip › Supplementary_Tables.pdf]

Supplementary Table S1. Seven-day SBP target achievement according to hematoma location

Hematoma location	Patients, n	Target achieved, n (%)
Lobar ICH	21	7 (33.3)
Deep ICH	48	20 (41.7)
Cerebellar ICH	6	0 (0)
Brainstem ICH	11	4 (36.4)
Unclassified	10	5 (50.0)
Total	96	36 (37.5)

Target SBP achievement was defined as a maximum recorded SBP below 140 mmHg in each of six consecutive 24-hour intervals from 24 to 168 hours after ICH onset. In an exploratory comparison between patients with lobar and deep ICH, no statistically significant difference in target achievement was observed (Fisher's exact test, $p = 0.598$; odds ratio, 0.70; 95% confidence interval, 0.24–2.05). ICH, intracerebral hemorrhage; SBP, systolic blood pressure.

Supplementary Table S2. Interval-specific patient counts and SBP target achievement rates used in the interrupted time-series analysis.

Time interval (six-month)	Study period	Number of eligible patients	Number achieving target SBP	Number not achieving target SBP	Target SBP achievement rate (%)
1. March 2019 – August 2019	Pre-intervention	11	3	8	27.3
2. September 2019 – February 2020	Pre-intervention	6	1	5	16.7
3. March 2020 – August 2020	Pre-intervention	4	1	3	25.0
4. September 2020 – February 2021	Pre-intervention	5	1	4	20.0
5. March 2021 – August 2021	Pre-intervention	10	2	8	20.0
6. September 2021 – February 2022	Pre-intervention	10	1	9	10.0
7. March 2022 – August 2022	Post-intervention	4	2	2	50.0
8. September 2022 – February 2023	Post-intervention	7	5	2	71.4
9. March 2023 – August 2023	Post-intervention	4	2	2	50.0
10. September 2023 – February 2024	Post-intervention	7	3	4	42.9
11. March 2024 – August 2024	Post-intervention	10	5	5	50.0
12. September 2024 – February 2025	Post-intervention	18	10	8	55.6
**Pre-intervention total (intervals 1–6)**		**46**	**9**	**37**	**19.6**
**Post-intervention total (intervals 7–12)**		**50**	**27**	**23**	**54.0**
**Overall (12 intervals)**		**96**	**36**	**60**	**37.5**

The 72-month study period (March 2019 – February 2025) was aggregated into 12 consecutive six-month intervals (6 before and 6 after protocol implementation); these intervals, not individual months, were the units of the interrupted time-series analysis. The protocol was implemented in March 2022, the first month of interval 7.

Target SBP achievement: maintenance of systolic blood pressure below 140 mmHg from 24 h to day 7 after onset. Achievement rate = number achieving target SBP / number of eligible patients × 100. The numbers achieving and not achieving the target in each interval formed the binomial outcome. SBP, systolic blood pressure.

Supplementary Table S3. Regression coefficients and effect estimates from segmented binomial logistic regression.

Parameter	Main model					Season-adjusted model				
	β	SE	OR	95% CI	P value	β	SE	OR	95% CI	P value
Intercept	−0.864	0.735	0.42	0.10–1.78	0.240	−0.868	0.737	0.42	0.10–1.78	0.239
Pre-intervention time trend (per interval)	−0.161	0.196	0.85	0.58–1.25	0.410	−0.165	0.202	0.85	0.57–1.26	0.413
**Immediate level change after intervention a**	**2.306**	**1.038**	**10.03**	**1.31–76.78**	**0.026**	**2.323**	**1.060**	**10.21**	**1.28–81.48**	**0.028**
Change in slope after intervention (per interval)	0.116	0.258	1.12	0.68–1.86	0.654	0.116	0.258	1.12	0.68–1.86	0.652
Post-intervention trend (per interval) b	−0.046	0.167	0.96	0.69–1.33	0.785	−0.049	0.172	0.95	0.68–1.33	0.776
Seasonal term (September–February vs March–August)	—	—	—	—	—	0.040	0.493	1.04	0.40–2.74	0.935
Residual deviance (df)	*1.78 (8)*					*1.77 (7)*				
Pearson χ^2 (df); dispersion	*1.74 (8); 0.22*					*1.73 (7); 0.25*				
AIC	*36.90*					*38.90*				

Segmented binomial logistic regression (binomial distribution, logit link) fitted to the numbers of patients achieving and not achieving the target SBP in each of 12 six-month intervals (grouped binomial data; n = 96), so that each interval contributed to the likelihood in proportion to its number of eligible patients. Model: logit(p) = β0 + β1·time + β2·intervention + β3·time after intervention (+ β4·season).

β, regression coefficient on the log-odds scale; OR = exp(β). 95% CIs were calculated by the Wald method [exp(β ± 1.96 × SE)]; P values are from Wald z tests. Time unit = one six-month interval.

a Time after intervention coded 0 in the first post-intervention interval (March–August 2022) and 1–5 thereafter; the OR represents the level change in the first interval exposed to the protocol relative to the projected pre-intervention trend. With time after intervention coded 1–6 (identical model fit), the OR is 8.94 (95% CI 1.21–66.03; P = 0.032), referring to a point extrapolated back to the last pre-intervention interval. Profile-likelihood 95% CI for the level change (main model): 1.44–90.23; likelihood-ratio test P = 0.019.

b Linear combination of the pre-intervention trend and the change in slope (β1 + β3); SE from the model covariance matrix.

Adding the seasonal term did not improve model fit (likelihood-ratio test P = 0.935); this does not establish the absence of seasonality. AIC, Akaike information criterion; CI, confidence interval; df, degrees of freedom; OR, odds ratio; SBP, systolic blood pressure; SE, standard error.

Supplementary Table S4. Model diagnostics and sensitivity analyses of the interrupted time-series analysis.

A. Model diagnostics

Item	Main model	Season-adjusted model	Comment
Number of time intervals	12 (6 pre, 6 post)	12 (6 pre, 6 post)	Six-month intervals; 96 patients
Error distribution and link function	Binomial; logit	Binomial; logit	Grouped data: achieved / not achieved per interval
Residual deviance (df)	1.78 (8)	1.77 (7)	
Residual degrees of freedom	8	7	
Pearson χ^2 statistic	1.74	1.73	
Dispersion estimate (Pearson χ^2/df)	0.22	0.25	< 1: no evidence of overdispersion
Lag-1 autocorrelation of Pearson residuals	−0.17	−0.18†	Approximate 95% bounds ±0.57
Ljung–Box test, lag 1	Q = 0.45; P = 0.502	Q = 0.50; P = 0.480†	
Ljung–Box test, lag 2	Q = 5.24; P = 0.073	Q = 5.25; P = 0.073†	
Durbin–Watson-type statistic	2.28	2.30†	≈ 2 if no lag-1 autocorrelation
Autocorrelation-specific adjustment	None	None	No ARIMA, Newey–West or other correction
Expected cell counts < 5	18 of 24	—	χ^2 approximations to be interpreted with caution

B. Sensitivity analyses: immediate level change after intervention

Analysis	OR (95% CI)	P value	Comment
Main model (reference)	10.03 (1.31–76.78)	0.026	Wald CI; Wald z test
Main model, profile likelihood	10.03 (1.44–90.23)	0.019	Profile-likelihood CI; likelihood-ratio test
Season-adjusted segmented binomial logistic regression	10.21 (1.28–81.48)	0.028	Season: September–February vs March–August
Firth penalized logistic regression	8.73 (1.36–70.56)	0.022	Profile penalized-likelihood CI and test
Alternative coding of time after intervention (1–6)	8.94 (1.21–66.03)	0.032	Identical fit; refers to last pre-intervention interval
Quasibinomial model (dispersion 0.22)	10.03 (3.88–25.92)	0.001	Not used for inference (dispersion < 1)
Leave-one-interval-out analysis (12 refits)	Range 6.04–13.18	Range 0.022–0.138	OR > 1 in 12/12; 95% CI included 1 in 3/12

Overdispersion was assessed by the ratio of the Pearson χ^2 statistic (and residual deviance) to the residual degrees of freedom. Residual autocorrelation was assessed by the autocorrelation function of the time-ordered Pearson residuals and Ljung–Box tests; with only 12 residuals, these tests have low power, and non-significant results do not demonstrate the absence of autocorrelation.

Firth regression (Jeffreys-prior penalty) reduces small-sample bias; profile penalized-likelihood CIs were obtained by direct maximization of the penalized log-likelihood. Leave-one-interval-out: the main model was refitted 12 times, omitting one interval each time.

† Calculated additionally for this table; not reported in the original results workbook. No previously reported value was changed. Analyses: R version 4.3.3 (brglm2 version 0.9.2). CI, confidence interval; df, degrees of freedom; OR, odds ratio.
